# Pelvic Digit: A Silent Guest in the Body

**DOI:** 10.7759/cureus.114744

**Published:** 2026-08-18

**Authors:** Sehrish Mehreen Rind, Devendra Kumar, Shoaib Ahmad Baig, Hanee Valiyakath, Soumya Hareendranath, Mahmoud Raheem Heidous

**Affiliations:** 1 Clinical Imaging Department, Al Wakra Hospital, Hamad Medical Corporation, Al Wakrah, QAT

**Keywords:** bony outgrowths, congenital pelvic rib, pain of hip, pelvic finger, pelvic rib

## Abstract

Pelvic digit is a rare congenital anomaly characterized by a finger-like bony structure near the pelvic bone, often discovered incidentally on imaging. Although typically asymptomatic, it may mimic other pathologies when symptoms arise. We report two cases: Case A involved a 14-year-old boy with post-traumatic foot pain, where imaging revealed a pelvic digit initially suspected to be an avulsion fracture; and Case B concerned a 40-year-old man with unrelated back pain, in whom the pelvic digit was found incidentally during imaging workup. In both cases, computed tomography and magnetic resonance imaging confirmed the diagnosis. Differentiating pelvic digit from conditions such as heterotopic ossification, avulsion fractures, and osteochondroma is critical to avoid unnecessary intervention. Awareness of this rare entity can prevent misdiagnosis, especially in patients presenting with chronic hip pain or palpable bony masses.

## Introduction

Pelvic digit, also referred to as a pelvic rib or pelvic finger, is a rare congenital anomaly characterized by a finger- or rib-like osseous projection located adjacent to the pelvic bone [1]. These bony structures are thought to arise from a developmental failure in mesenchymal segmentation, leading to ectopic ossification within soft tissues near the ilium or acetabulum [2]. Although typically asymptomatic, pelvic digits are often discovered incidentally on routine radiographs performed for unrelated reasons. However, due to their unusual location and appearance, they may be mistaken for more common pathological entities such as avulsion fractures, myositis ossificans, heterotopic ossification, or even osteochondromas [3]. This diagnostic confusion may result in unnecessary investigations, referrals, or even surgical intervention.

Awareness of this entity, alongside careful assessment using advanced imaging modalities such as computed tomography (CT) and magnetic resonance imaging (MRI), is essential to avoid misinterpretation. The presence of well-corticated margins, pseudoarticulations, and continuity with soft-tissue structures are typical features that distinguish pelvic digits from true pathology [4,5]. We present two cases of pelvic digit: one discovered incidentally during imaging for unrelated back pain, and another initially misinterpreted as an avulsion injury following trauma. These cases illustrate the diagnostic challenge and underscore the importance of radiologic recognition to prevent overtreatment.

## Case presentation

Case 1

**Figure 1:**
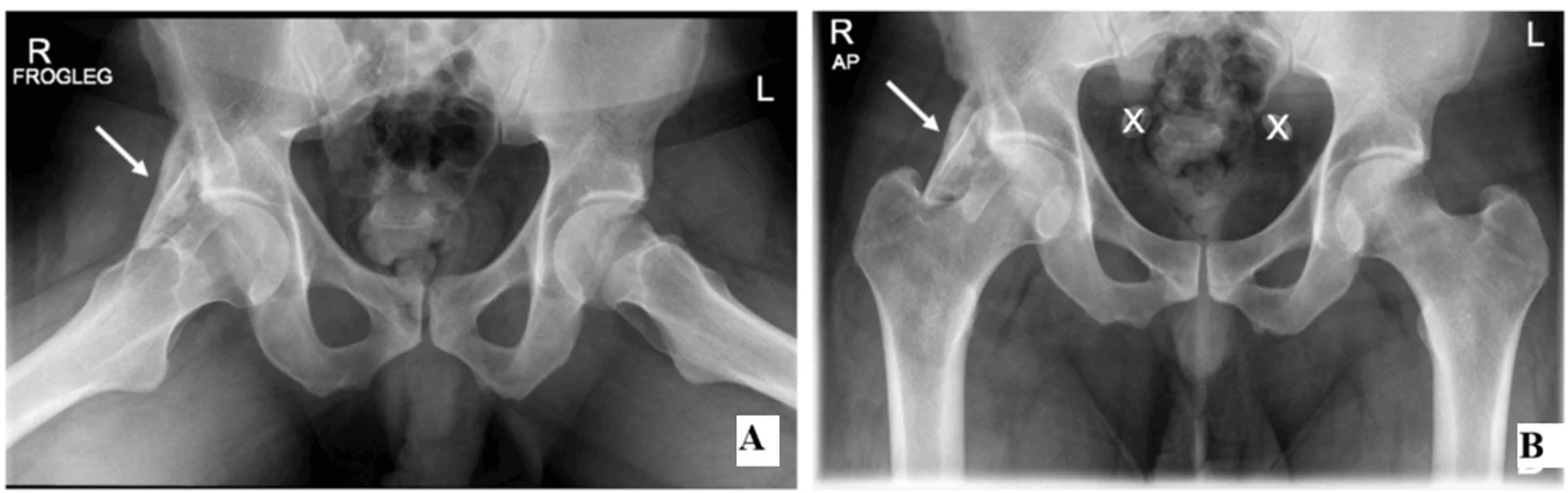
Plain radiographs of the pelvis, showing a well-defined bony bar anterosuperior to the right hip joint with pseudoarthrosis with the right anterior inferior iliac spine (pathognomonic of pelvic digit). (A) Frog view. (B) AP view AP: anteroposterior

**Figure 2:**
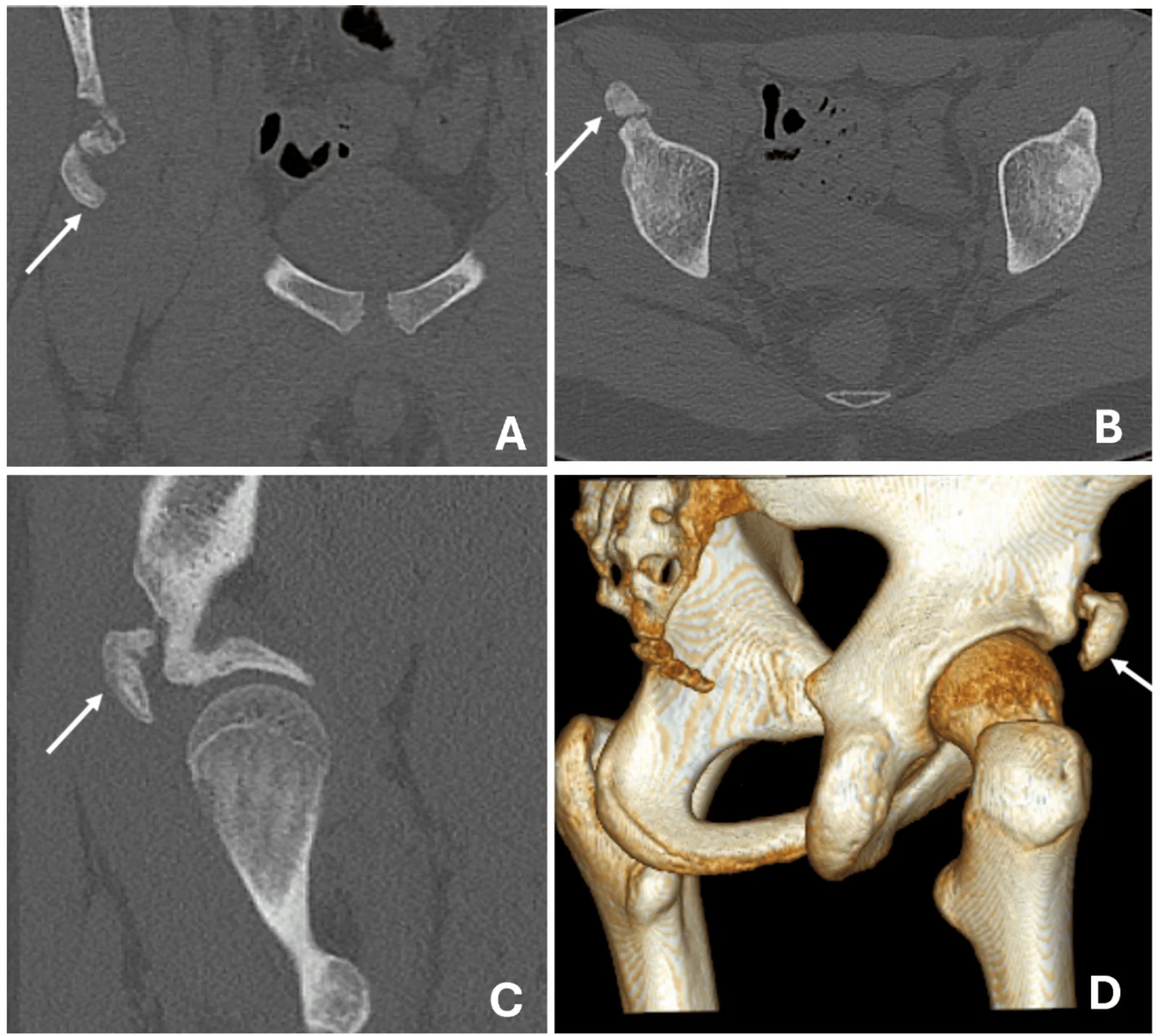
Noncontrast CT of the pelvis. Axial (A), coronal (B), and sagittal (C) planes and 3D VR (D) image confirming the congenital pelvic digit configuration of bony protuberance with changes of pseudoarthrosis with right anterior inferior iliac spine CT: computed tomography; 3D: three dimensional; VR: virtual reality

A 14-year-old boy presented for a mildly displaced fracture involving the proximal phalanx of the third toe while playing soccer. On examination, he had localized tenderness, a bruise, and swelling on the foot. Post-reduction and buddy strapping, he was discharged and advised to follow up in the OPD after two weeks. Plain radiographs of the pelvis and right femur were done on orthopedic follow-up due to a complaint of right hip pain without any restriction of movement. There was a linear bony structure found adjacent to the right inferior iliac spine, which was assumed to be an avulsion fracture (Figure 1), keeping in view the history of previous trauma. CT of the pelvis was performed due to the discrepancy between the large well-corticated bone fragment and minimal clinical symptoms to assess for an avulsion injury and the nature of the bone fragment. CT scan with three-dimensional bony (Figure 2) reconstruction showed a well-corticated “L”-shaped bone fragment of about 2.4 cm × 1.5 cm along the right inferior iliac spine with a suggestion of a pointed opposing surface with iliac bone. There was no obvious callus formation. The surrounding soft tissues were unremarkable. The rectus femoris muscle attachment and the right hip joint were normal. He was managed conservatively by the orthopedic surgeon and advised for physiotherapy.

Case 2

**Figure 3:**
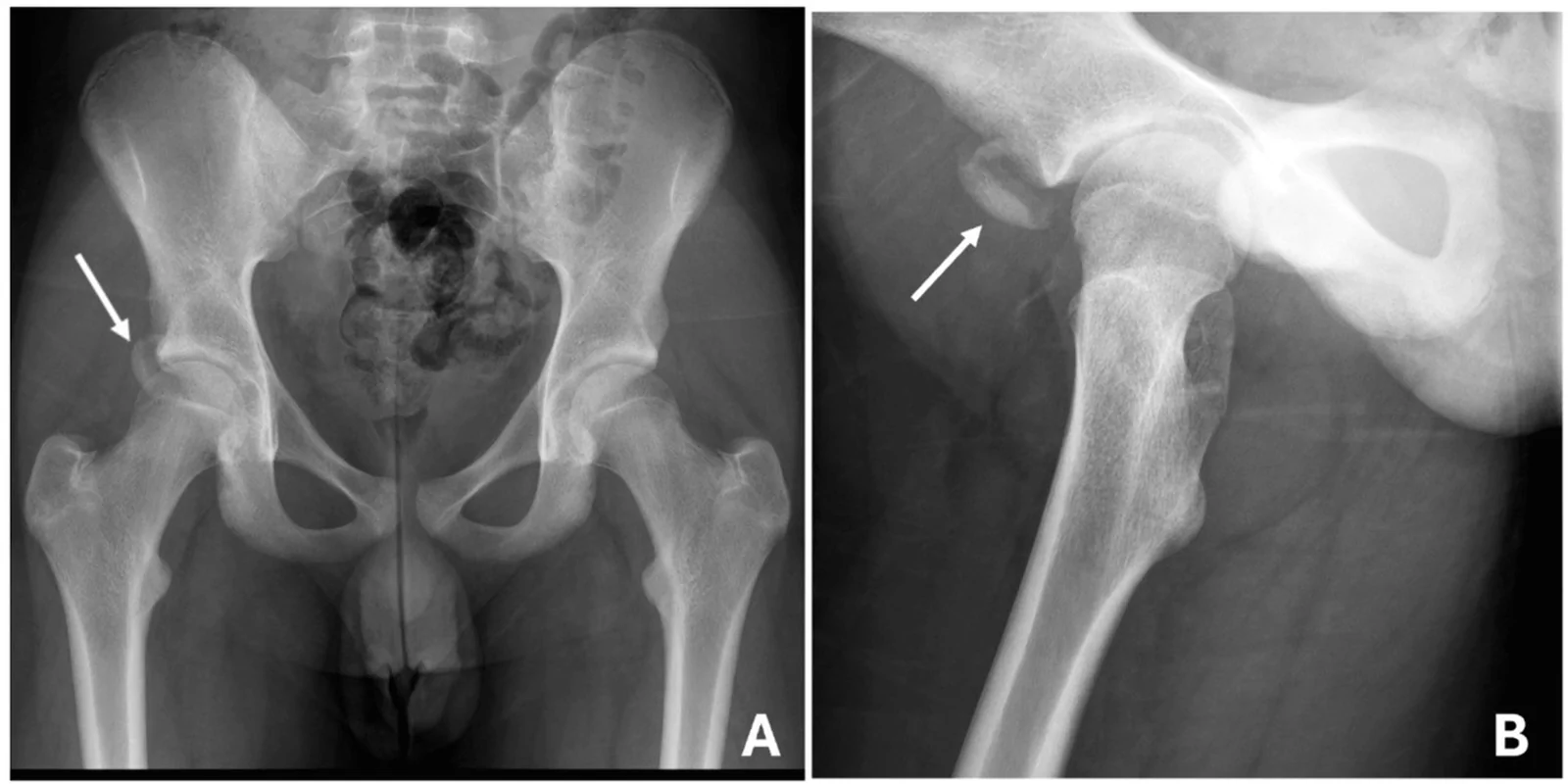
Plain radiographs of the pelvis AP (A) and oblique right hip joint (B) showing well-corticated "L"-shaped bony protuberance from right iliac bone, which extend toward the hip joint, a characteristic of pelvic digit AP: anteroposterior

**Figure 4:**
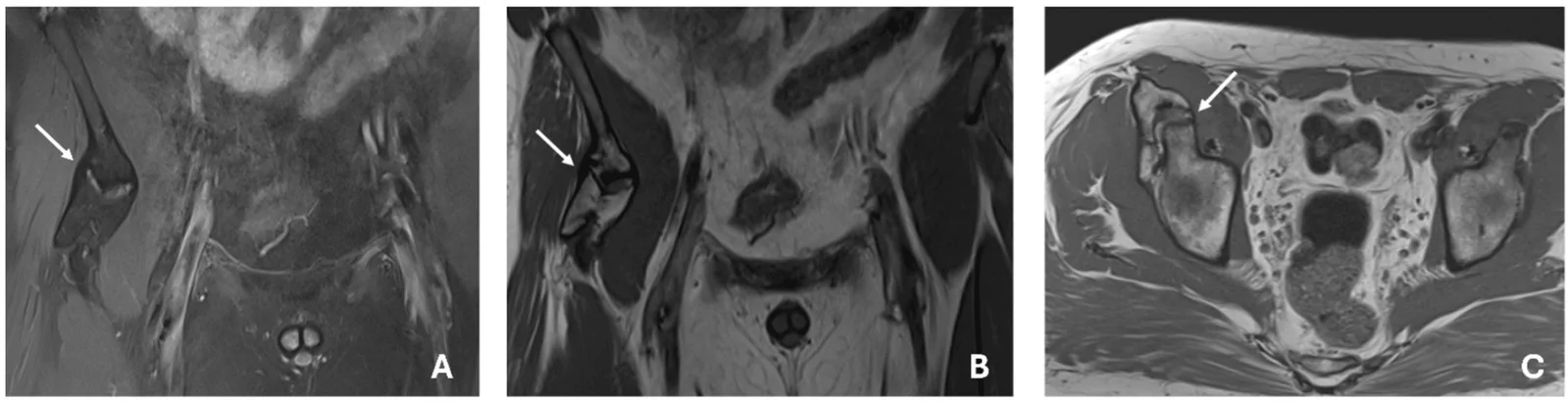
T2 coronal fat-saturated (A), T1 coronal (B), and T1 axial (C) MRI images confirming the bony fragment projecting from the right anterior inferior iliac spine with pseudoarthrosis, pelvic digit MRI: magnetic resonance imaging

A 40-year-old male patient with a complaint of lower back pain presented in OPD and was advised for lumbosacral spine and sacroiliac joint radiographs. Apart from routine spondylotic changes in the lower lumbar vertebrae, an incidental detection of an elongated bony bar was seen adjacent to the right anteroinferior iliac spine with changes of pseudoarthrosis and suspicion of an old avulsion injury of the rectus femoris from the anterior inferior iliac spine (Figure 3). MRI of the right hip joint was performed, which confirmed the pseudoarthrosis of a well-corticated finger-like bony fragment with the right anteroinferior iliac spine projecting into the soft tissue (Figure 4). There was no evidence of marrow edema and soft tissue inflammation. The right hip joint and the femoral head are normal. The patient was clinically asymptomatic; hence, no further actions were taken. Only management for his back pain was planned accordingly.

## Discussion

In this report, we describe two cases of pelvic digit, a rare congenital anomaly characterized by rib- or finger-like bony structures adjacent to the pelvic skeleton. One case was identified incidentally, whereas the other involved post-traumatic symptoms. In both cases, cross-sectional imaging was essential in confirming the diagnosis and avoiding misinterpretation as a pathological lesion.

Pelvic digit is frequently misdiagnosed due to its resemblance to avulsion fractures, heterotopic ossification, or osteochondroma on plain radiographs. Whereas avulsion injuries typically exhibit irregular, non-corticated margins and a clear trauma history, pelvic digits are well-corticated and may present with a pseudoarticulation at the base. Such radiographic confusion may lead to unnecessary clinical investigations or interventions, as previously reported in individual case studies and reviews [5,6].

Advanced imaging modalities are critical in distinguishing pelvic digit from other entities. CT provides excellent detail of cortical continuity and marrow structure [7], whereas MRI assists in evaluating adjacent soft tissues and excluding reactive or neoplastic changes. These imaging features allow accurate identification of a pelvic digit as a benign anatomical variant, particularly in symptomatic or post-trauma settings.

The pathogenesis of pelvic digit is linked to embryological errors during mesodermal development. Normally, costal processes in the pelvic region undergo programmed apoptosis, but failure of this process may allow rib-like ossification to persist near the iliac bone or pelvic wall. Alternative theories have proposed accessory ossification centers near the anterior superior iliac spine or displaced rib mesenchyme as potential origins [3]. These anomalies most commonly occur unilaterally and are typically solitary, although bilateral and multiple cases have been documented [2].

Although pelvic digit is usually asymptomatic, it can become clinically relevant in the context of trauma, soft tissue irritation, or impingement on adjacent structures. When pain or mechanical symptoms arise, especially without a clear cause, clinicians must consider this anomaly in the differential diagnosis. Misdiagnosis may lead to unwarranted procedures such as biopsy, surgical resection, or orthopedic referral [8].

Our report is subject to limitations. First, it represents only two cases, which may not capture the full spectrum of pelvic digit presentation. Second, histological confirmation was not obtained, as the imaging findings were definitive and consistent with prior reports. Finally, both cases originate from a single institution, which may limit generalizability. Nevertheless, the consistency of imaging across cases and the alignment with published literature strengthen the validity of our observations.

These cases emphasize the importance of radiological awareness and pattern recognition in distinguishing pelvic digit from pathology. Clinicians and radiologists should consider this entity in the differential diagnosis of well-corticated pelvic bone projections, particularly those with pseudoarticulation and no evidence of soft tissue inflammation.

## Conclusions

The pelvic digit is a benign congenital anomaly that can easily be mistaken for more serious pathologies if not properly recognized. Awareness of its characteristic imaging features assists clinicians in avoiding unnecessary interventions. Accurate diagnosis relies heavily on imaging modalities such as CT and MRI, which provide detailed insights into cortical and medullary continuity of the ossified projection. Recognizing and understanding its imaging features have significance in differentiating this entity from other differentials.

## References

[1] Moreta-Suárez J, de Ugarte-Sobrón OS, Sánchez-Sobrino A, Martínez-De Los Mozos JL (2012). The pelvic digit: a rare congenital anomaly as a cause of hip pain. J Orthop Case Rep.

[2] Goyen M, Barkhausen J, Markschies NA, Debatin JF (2000). The pelvic digit - a rare developmental anomaly. A case report with CT correlation and review of the literature. Acta Radiol.

[3] Maegele M (2009). Pelvic digit as a rare cause of chronic hip pain and functional impairment: a case report and review of the literature. J Med Case Rep.

[4] Van Breuseghem I (2006). The pelvic digit: a harmless "eleventh" finger. Br J Radiol.

[5] Malla B, Reddy BR (2024). A rare symptomatic pelvic rib - a case report. J Orthop Case Rep.

[6] Keser S, Bayar A, Savranlar A (2003). Pelvic digit: a case report with reference to the differential diagnosis of pelvis abnormalities. [Article in Turkish]. Acta Orthop Traumatol Turc.

[7] McGlone BS, Hamilton S, FitzGerald MJ (2000). Pelvic digit: an uncommon developmental anomaly. Eur Radiol.

[8] Hamilton S (1985). Pelvic digit. Br J Radiol.

